# *Trypanosoma brucei* Modifies the Tsetse Salivary Composition, Altering the Fly Feeding Behavior That Favors Parasite Transmission

**DOI:** 10.1371/journal.ppat.1000926

**Published:** 2010-06-03

**Authors:** Jan Van Den Abbeele, Guy Caljon, Karin De Ridder, Patrick De Baetselier, Marc Coosemans

**Affiliations:** 1 Department of Animal Health, Unit of Veterinary Protozoology, Institute of Tropical Medicine Antwerp (ITM), Antwerp, Belgium; 2 Unit of Cellular and Molecular Immunology, Vrije Universiteit Brussel (VUB), Brussels, Belgium; 3 Department of Molecular and Cellular Interactions, VIB, Ghent, Belgium; 4 Department of Parasitology, Unit of Entomology, Institute of Tropical Medicine Antwerp (ITM), Antwerp, Belgium; National Institute of Allergy and Infectious Diseases, United States of America

## Abstract

Tsetse flies are the notorious transmitters of African trypanosomiasis, a disease caused by the *Trypanosoma* parasite that affects humans and livestock on the African continent. Metacyclic infection rates in natural tsetse populations with *Trypanosoma brucei*, including the two human-pathogenic subspecies, are very low, even in epidemic situations. Therefore, the infected fly/host contact frequency is a key determinant of the transmission dynamics. As an obligate blood feeder, tsetse flies rely on their complex salivary potion to inhibit host haemostatic reactions ensuring an efficient feeding. The results of this experimental study suggest that the parasite might promote its transmission through manipulation of the tsetse feeding behavior by modifying the saliva composition. Indeed, salivary gland *Trypanosoma bruce*i-infected flies display a significantly prolonged feeding time, thereby enhancing the likelihood of infecting multiple hosts during the process of a single blood meal cycle. Comparison of the two major anti-haemostatic activities i.e. anti-platelet aggregation and anti-coagulation activity in these flies versus non-infected tsetse flies demonstrates a significant suppression of these activities as a result of the trypanosome-infection status. This effect was mainly related to the parasite-induced reduction in salivary gland gene transcription, resulting in a strong decrease in protein content and related biological activities. Additionally, the anti-thrombin activity and inhibition of thrombin-induced coagulation was even more severely hampered as a result of the trypanosome infection. Indeed, while naive tsetse saliva strongly inhibited human thrombin activity and thrombin-induced blood coagulation, saliva from *T. brucei*-infected flies showed a significantly enhanced thrombinase activity resulting in a far less potent anti-coagulation activity. These data clearly provide evidence for a trypanosome-mediated modification of the tsetse salivary composition that results in a drastically reduced anti-haemostatic potential and a hampered feeding performance which could lead to an increase of the vector/host contact and parasite transmission in field conditions.

## Author Summary

Human African Trypanosomiasis, or sleeping sickness, is a devastating parasitic disease that is fatal if left untreated. Infections are acquired via the bite of an obligate blood feeding fly, the tsetse fly, that is exclusively present on the African continent. In this insect vector, the trypanosome parasite has a complex development ending in the salivary glands. In this experimental study we demonstrate that the *Trypanosoma brucei* parasites change the composition of the tsetse fly saliva making it less efficient to keep the blood fluid at the biting site in the mammalian host. This results in a more difficult blood feeding process and favors the fly biting activity on multiple hosts, thereby promoting the survival and circulation of the parasite within the natural host population. These findings give us a better understanding of how trypanosome infections in the human population can be maintained given the fact that only very few tsetse flies are actually carrying the parasite.

## Introduction

Tsetse flies (*Diptera*: *Glossinidae*) are obligate blood feeding insects that are important disease vectors given their involvement in the transmission of different pathogenic trypanosome species that cause human sleeping sickness and livestock trypanosomiasis in Africa. Trypanosomes of the *Trypanosoma brucei* group – including the two human-pathogenic subspecies *T. b. gambiense* and *T. b. rhodesiense* – have to go through a complex developmental cycle in the alimentary tract and salivary glands of the tsetse fly [1]. The salivary gland is the tissue in which *T. brucei* parasites undergo the final developmental phase, i.e. a continuous cycle of multiplication and cellular differentiation into the metacyclic form that is infective for the mammalian host [2]. Once this trypanosome population has been established in the salivary glands, it is continuously maintained at high density throughout the remaining life span of the tsetse fly.

In the naive salivary gland micro-environment, saliva components are present that enhance the infection onset upon trypanosome inoculation in the host skin [3]. Other constituents are essential for the hematophagous behavior of the tsetse fly by counteracting host responses such as vasoconstriction, platelet aggregation and coagulation reactions involving serine proteases such as thrombin [4]. Several compounds have been implicated in facilitating blood feeding: a thrombin inhibitor [tsetse thrombin inhibitor (TTI)] [5], [6] and salivary apyrases [5′nucleotidase related protein, *Glossina morsitans morsitans* salivary gland protein 3 (Sgp3)] including at least one with fibrinogen receptor (GPIIb/IIIa) antagonistic properties (5′Nuc) [7]. Other abundant salivary components include putative endonucleases [tsetse salivary gland proteins 1 and 2 (Tsal1 and Tsal2)] [8], putative adenosine deaminases [tsetse salivary gland growth factors 1 and 2 (TSGF-1 and TSGF-2)] [9] and an antigen5-related allergen [tsetse Antigen5 (TAg5)] [10]. However, there is no information on the importance of these major tsetse saliva proteins in their interplay with the trypanosome life cycle.

To date, a growing number of studies demonstrate the ability of vector-borne parasites to alter phenotypic traits of their insect vectors in a way that increases vector-host contact frequency and hence increases the probability of parasite transmission [11], [12]. This type of parasite-induced modulation of the vector physiology and feeding behavior has already been documented for the *Leishmania*-sandfly model [13], the *Plasmodium*-mosquito model [14]–[16] and other pathogen-vector models [reviewed in [17], [18]]. A recurrent feature of infected vectors is a modified feeding behavior that results from the physical obstruction of the alimentary tract and interference with mechanoreceptors that are required to regulate the blood meal uptake. Indeed, *Leishmania* promastigotes produce a secretory gel, mainly composed of a filamentous proteophosphoglycan that blocks the foregut and impairs the phagoreceptors, thereby reducing the arthropod feeding efficiency [19]. Similarly, a proportion of plague-transmitting fleas display obstructed proventiculi as a result of *Yersinia* biofilm surrounded by an extracellular matrix [20]. In the tsetse fly-trypanosome interaction, mouthpart obstruction and interference with labral mechanoreceptors has been documented upon infection with *Dutonella* and *Nannomonas* subgenera of *Trypanosoma* (*T. congolense* and *T. vivax*) that form rosettes and colonize the tsetse fly labrum [21]–[26]. However, limited and contradictory data have been reported on the feeding behavior of tsetse flies infected with *T. brucei* parasites (including the human pathogens) which belong to the *Trypanozoon* subgenus and display a different developmental cycle in the vector than *T. congolense* and *T. vivax* [1], [2]. Jenni *et al.* observed a more frequent probing behavior of *T. brucei* infected tsetse flies and hypothesized that this resulted from physical interference of trypanosomes with the function of the labral mechanoreceptors [27]. However, other experimental results suggested that *T. brucei* parasites in the salivary glands did not significantly alter the tsetse feeding [22], [28].

In this study, we investigated whether *T. brucei* parasites alter the tsetse fly blood feeding behavior in a way that would favor parasite transmission within the mammalian host population. Next, we determined the impact of a *T. brucei* salivary gland infection on the saliva composition and the biological activities related to anti-haemostasis. The obtained data provide evidence that the trypanosome parasites drastically modulate the tsetse salivary composition and anti-haemostatic activity resulting in an alteration of the feeding behavior that favors parasite transmission.

## Results

### Effect of salivary gland infection on tseste feeding efficiency

The feeding efficiency of salivary gland infected (SG^+^) tsetse flies (*n* = 50) was compared to that of controls that did not develop a salivary gland infection (SG^-^, *n* = 48) upon feeding on a *Trypanosoma brucei brucei* AnTAR1 parasitemic mouse. As a read-out, two variables were measured: (i) the time necessary to obtain a full blood meal including the probing behavior that precedes the actual blood ingestion and (ii) the size (mass) of the blood meal. Despite a considerable variability in both experimental groups, the blood meal acquisition was significantly slower (*p<*0.05, Table 1) for SG^+^ flies (267±23 s.) than for SG^-^ flies (210±16 s.), especially resulting from a prolonged probing behavior (visual observation). No differences in ingested blood masses were observed (*p* = 0.83).

**Table 1:** Feeding performance of tsetse flies.

	SG-non infected	SG-infected	*p*-value
**Time for feeding** ± s.e.m. (n)	210 s±16 (48)	267 s±23 (50)	<0.05
**Size of blood meal** ± s.e.m. (n)	19.7 mg±1.7 (48)	19.2 mg±1.8 (48)	>0.05

Feeding efficiency (feeding time and blood meal size) of uninfected (SG^-^) and *T. brucei* salivary gland infected (SG^+^) tsetse flies. The reported feeding time is the total time that individual flies needed for the pre-feeding probing and the actual feeding. Blood meal sizes were determined by measuring the individual fly masses before and immediately after blood meal acquisition.

(n) =  number of samples.

SG  =  salivary gland.

### Effect of salivary gland infection on salivary gene transcription and translation

**Figure 1:**
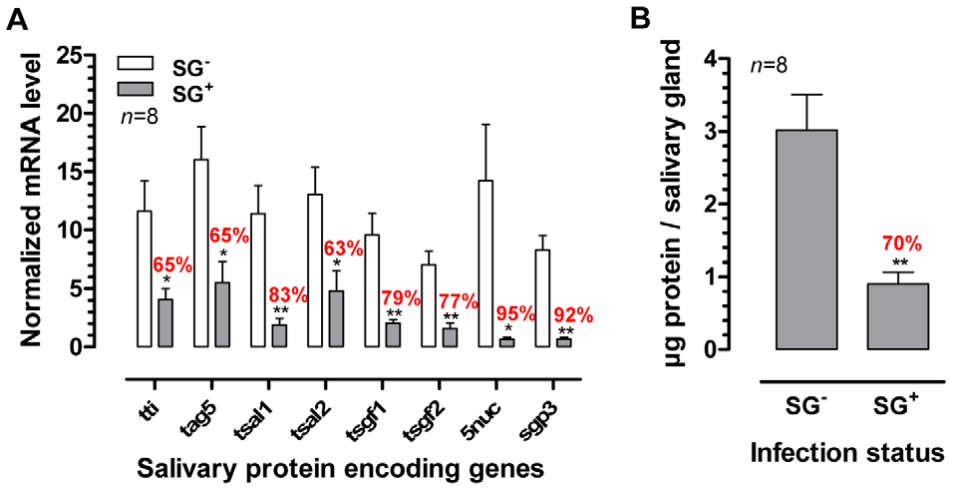
Effects of the *T. brucei* salivary gland infection on tsetse fly salivary protein expression. (**A**) RT-qPCR expression analysis of genes encoding the major tsetse saliva proteins (TTI, TAg5, Tsal1/2, TSGF1/2, 5′Nuc and Sgp3) in the salivary glands of *T. brucei* infected (SG^+^) and uninfected (SG^-^) flies. mRNA levels were normalized using both actin and tubulin as housekeeping genes. (**B**) Total protein content in the saliva of SG^+^ and SG^-^ tsetse flies. The bars denote means and SEM-values observed among 8 independent samples tested in each group. The ^*^ and ^**^ denotes a significant difference between the two experimental groups, respectively with *p*<0.05 and *p*<0.01.

**Figure 2:**
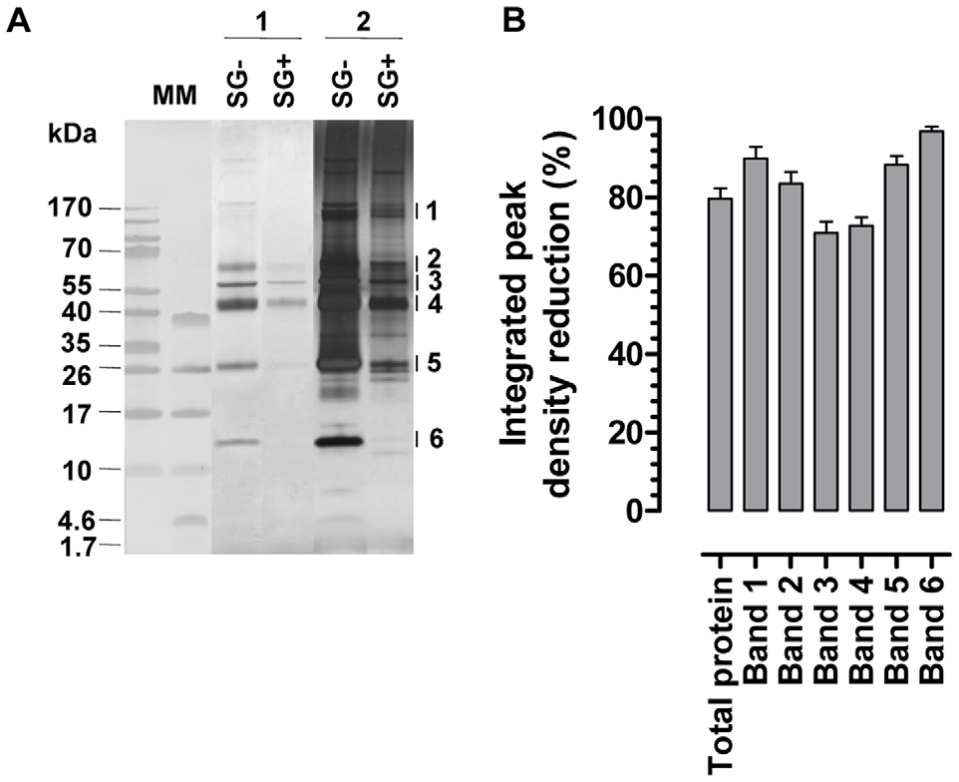
Effects of the *T. brucei* salivary gland infection on the tsetse fly salivary protein composition. (**A**) Tricine-SDS-PAGE analysis on equal volumes of saliva harvested from *T. brucei* infected (SG^+^) and uninfected (SG^-^) flies. Proteins in de gel were fixed and stained with either coomassie dye (lanes section 1; 6 µl/lane saliva obtained from 3 gland pairs harvested in 30 µl) or silver salts (lanes section 2; 2 µl/lane saliva obtained from 3 gland pairs harvested in 30 µl). The major protein bands (1–6) that were subjected to densitometric analysis are indicated on the right side of the gel. Molecular markers (MM) are respectively the PageRuler 100–10 kDa and Spectra 40–1.7 kDa prestained protein ladder. (**B**) Percentages reduction of integrated peak densities of the entire protein composition and the individual protein bands (1–6; see panel A) in SG^+^ saliva as revealed by densitometric comparison of SG^-^ and SG^+^ Coomassie stained protein profiles. The 6 major protein bands are anticipated to represent respectively Sgp3, 5′Nuc, TSGF1&2, Tsal1&2, TAg5 and GE-1&2 [30], [38].

The presence of a *T. b. brucei* infection in the salivary glands significantly compromised (*p*<0.05) the expression of genes that encode the major *G. m. morsitans* saliva proteins (Figure 1A). Expression levels were decreased by 63% (*tsal 2*) up to 95% for the *5*′*nuc* apyrase gene (Figure 1). In two independent experiments, threshold cycle values for actin and tubulin housekeeping genes did not significantly change as a result of the SG^+^ infection status. Concomitant to the reduced transcription of the major saliva genes, the saliva of SG^+^ flies contained 70% less protein (*p*<0.01) as compared to the SG^-^ flies (0.9±0.2 versus 3.0±0.5 µg per salivary gland, Figure 1B). A more detailed analysis of the SG^-^ and SG^+^ saliva composition was performed using Tricine-SDS-PAGE (Fig. 2A) combined with either Coomassie (Fig. 2A, lanes section 1) or Silver based staining methods (Fig. 2A, lanes section 2). Densitometry analysis of the Coomassie stained protein profiles revealed a generalized reduction of 70–97% in protein band intensities for SG^+^ saliva samples (Fig. 2B). In addition, several protein and peptide bands that are visible in the SG^-^ saliva profiles upon silver staining, are no longer detectable in SG^+^ saliva. Western blot analysis using anti-*T. b. brucei* infectome immune serum could not detect the appearance of trypanosome-derived components in SG^+^ saliva.

### Effect of salivary gland infection on salivary biological activities

**Figure 3:**
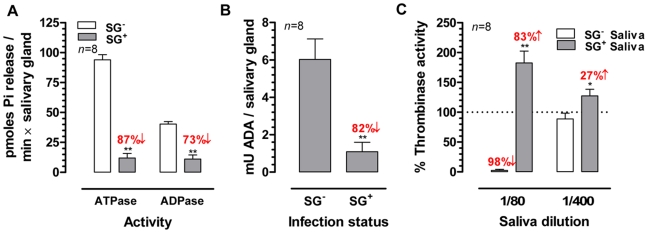
Effects of the *T. brucei* salivary gland infection on the biological activities of tsetse fly saliva. (**A**) Apyrase (ATPase and ADPase) activity in the SG^+^ and SG^-^ saliva determined using a P*_i_*-release assay. (**B**) Adenosine deaminase activity expressed as milliunits (mU) per salivary gland in SG^+^ and SG^-^ saliva. (**C**) Thrombin-inhibitory activity of SG^+^ and SG^-^ tsetse fly saliva (1/80 and 1/400) determined in a pNA-release assay using 750 nM thrombin chromogenic substrate and 500 mU/ml human thrombin. The bars represent means and SEM-values for eight independent samples tested in each group. The percentages activity increase and decrease are indicated above the respective bars. The ^*^ and ^**^ denotes a significant difference between the two experimental groups, respectively with *p*<0.05 and *p*<0.01.

Different biological activities (apyrase, adenosine deaminase and anti-thrombinase) that were previously described or suggested to be present in tsetse saliva, were quantified in SG^-^ and SG^+^ samples. Based on the quantification of P*_i_*-release from the individual substrates ATP and ADP as read-out for apyrase (ATP diphosphohydrolase) activity, an approximate 5-fold reduction (*p*<0.01) in salivary apyrase was observed in trypanosome infected salivary glands (Figure 3A). For the adenosine deaminase activity that was present in the SG^-^ saliva at 6.0±1.0 mU/salivary gland, a similar reduction (82%, *p*<0.01) was observed in the SG^+^ flies exhibiting an activity of only 1.1±0.5 mU/salivary gland (Figure 3B). The thrombinase-inhibitory properties of tsetse fly saliva were assayed with respectively 1/80 and 1/400 dilutions. The 1/80 SG^-^ saliva dilutions almost completely inhibited the human thrombinase activity (assayed by the release of pNA from thrombin-specific substrate) at the concentration of 500 mU/ml (Figure 3C). In contrast, a significant increase (83%, *p*<0.01) in thrombinase activity was observed for the same SG^+^ saliva dilution, suggesting a potentiation of the thrombin enzymatic activity in the used assay conditions. For the 1/400 SG^+^ dilution, an increase could still be detected although less pronounced (27%, *p*<0.05). The enhancement of thrombinase activity by SG^+^ saliva did not depend on a trypanosome-derived enzyme with the same substrate-specificity, as saliva from SG^+^ flies by itself did not convert the chromogenic substrate (data not shown).

### Effect of salivary gland infection on salivary anti-haemostatic activities

**Figure 4:**
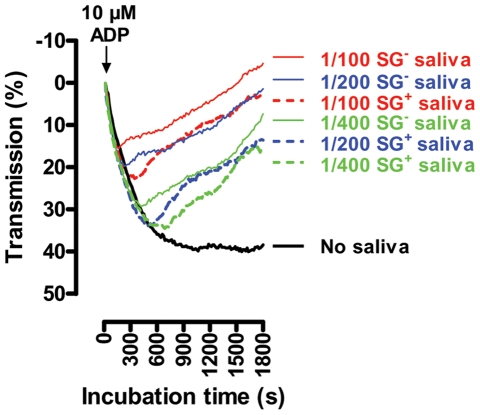
Effect of the *T. brucei* salivary gland infection on the anti-thrombotic properties of tsetse fly saliva: inhibition of 10 µM ADP-induced platelet aggregation by ½ serial SG^+^ and SG^-^ saliva dilutions (1/100 to 1/400). Platelet aggregation was observed as a reduced absorption (increase in transmission) at λ = 650 nm. These data are representative of 2 experiments using 4 independent biological samples per experimental group, tested in duplicate at the different dilutions.

**Figure 5:**
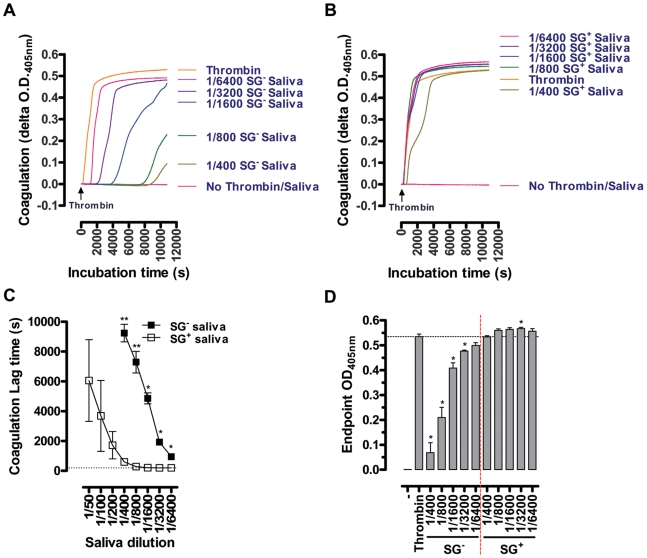
Effect of the *T. brucei* salivary gland infection on the anti-coagulant properties of tsetse fly saliva: inhibition of 25 mU/ml thrombin-induced coagulation by ½ serial SG^+^ and SG^-^ saliva dilutions (1/50 to 1/6400). Representative coagulation profiles for the 1/400 to 1/6400 SG^-^ (**A**) and SG^+^ samples (**B**) are depicted, illustrating the strong reduction of anti-coagulant activity in saliva upon salivary gland *T. brucei* infection. (**C**) Lag times before the coagulation onset, observed as a steep O.D._405 nm_ increase of the PPP, were determined as measure for anti-coagulant activity of the different SG^+^ and SG^-^ saliva dilutions. (**D**) Maximal coagulation responses (O.D._405 nm_) within a 3 hour reaction time. These data are compiled from 2 experiments using 4 independent biological samples per experimental group, tested in duplicate at the different dilutions.

The salivary anti-thrombotic and anti-coagulant activities were monitored in human plasma using respective *in vitro* read-out assays. The aggregation of platelets in human platelet rich plasma (PRP) supplemented with ½ serial SG^-^ and SG^+^ saliva dilutions (1/100–1/400) was analyzed in response to 10 µM ADP, revealing an approximate 3-fold reduction in anti-platelet aggregating capacity of SG^+^ saliva (Figure 4). Coagulation in human platelet poor plasma (PPP), induced by 25 mU/ml thrombin in the presence or absence of ½ serial SG^-^ (1/400–1/6400) and SG^+^ saliva dilutions (1/50–1/6400), revealed a striking decrease of anti-coagulant activity in tsetse fly saliva upon trypanosome infection. Indeed, while all tested SG^-^ saliva dilutions (1/400–1/6400) markedly increased the coagulation lag times (Figure 5A), all SG^+^ saliva dilutions from 1/400 downwards (1/800–1/6400) exerted negligible anti-coagulant activity (Figure 5B). Comparison of the coagulation lag times revealed a 16- to 32-fold reduction of anti-coagulant activity in SG^+^ as compared to SG^-^ saliva (Figure 5C). Moreover, thrombin did not induce maximal coagulation responses in PPP in the presence of the 1/400–1/6400 SG^-^ saliva samples within a 3 hour reaction time (Figure 5D), while endpoint O.D. values at 405 nm were even slightly higher when thrombin was incubated with 1/800–1/6400 SG^+^ saliva samples (Figure 5D). These slightly increased endpoint O.D. values did not result from clotting of salivary components, as no thrombin-induced coagulation was observed in SG^-^ and SG^+^ saliva (data not shown).

## Discussion

African trypanosomes including the human-infectious *Trypanosoma brucei* subspecies, exploit the obligate blood feeding behavior of tsetse flies (*Glossina* sp.) for their transmission. These tsetse fly vectors rely on a pool feeding strategy which involves the laceration of the skin with their proboscis and blood ingestion from a superficial lesion. Once the skin is pierced, the proboscis is often partially withdrawn before being thrust again at a slightly different angle to probe for suitable blood vessels and to enhance the blood pool formation [29]. During these events, about 4 µg of salivary proteins are inoculated at the bite site in order to neutralize the complex anti-haemostatic host reactions that would lead to blood clotting and vasoconstriction [30]. In the case of feeding on a parasitemic host, tsetse flies can acquire a trypanosome infection which depends on a complex sequence of differentiation and migration that ends in the insect salivary glands [1], [2]. Once the salivary glands are colonized by metacyclic *T. brucei* parasites (SG^+^), the tsetse fly can transmit parasites throughout its entire lifespan at each vector/host contact.

Despite the epidemiological importance, information on the impact of the salivary gland infection on the tsetse feeding behavior and trypanosome transmission is scanty and contradictory. While Moloo *et al.* did not observe significant feeding behavioral differences as a result of the SG^+^ status [22], Jenni *et al*. [27] reported that *T. brucei*-infected flies probed more frequently (2 to 3 fold increase) before feeding and subsequently fed more voraciously as compared to uninfected (SG^-^) flies. The authors suggested that these effects resulted from the association of some trypanosomes with labral mechanoreceptors that play a role in the feeding and gorging response, analogous to what was reported for *T. congolense* infected flies. Indeed, the increased probing activity of *T. congolense* infected *G. morsitans* flies [31] may be caused by physical interference of the parasite with phagoreceptors in combination with a reduced diameter of the tsetse labrum due to the presence of parasite rosettes [22]–[23], [32]. However, in contrast to *T. congolense*, *T. brucei* parasites never permanently colonize the tsetse fly mouthparts where the mechanoreceptors are localized [1], [2], which is not supportive for Jenni's hypothesis. In our study, we could confirm Jenni's observation that a *T. brucei* infection in tsetse fly salivary glands does significantly disturb the fly feeding behavior. Indeed, SG^+^ tsetse flies needed significantly longer times (>25% longer) to complete blood feeding due to a prolonged pre-feeding probing phase. Our experimental data clearly suggest that this altered feeding phenotype is the consequence of a changed protein content of the tsetse saliva due to the presence of a trypanosome infection, resulting in a much less potent anti-haemostatic activity. This reduced saliva production was confirmed using Tricine-SDS-PAGE, revealing a generalized suppression (70–97%) of all protein bands in tsetse saliva which was found to be associated with a severely reduced (63%–95%) transcription of the major tsetse fly salivary genes. Especially the *5*′*nuc* gene that encodes an important tsetse fly salivary apyrase with GPIIb/IIIa (fibrinogen receptor) antagonistic properties [7] and another putative apyrase gene (*sgp3*) were strongly suppressed (>90%) resulting in an overall 80% down regulation in the saliva apyrase [AT(D)Pase] activity. A similar phenomenon has been described for *Plasmodium* infected mosquitoes, where the salivary apyrase activity was reduced by three fold and which was also associated with prolonged probing times [14], [33]. Salivary apyrase activity underlies one of the major anti-haemostatic strategies in a blood feeding insect [34] given that these enzymes inhibit purinergic thrombocyte triggering by hydrolyzing ATP and ADP, haemostatic triggers that are released from injured cells and activated platelets [35]. As such, the reduced apyrase activity in the SG^+^ tsetse saliva seriously affected the normally powerful capacity to inhibit the blood platelet aggregation demonstrated in an *in vitro* aggregation studies using human platelets. The significant suppressed adenosine deaminase activity in the trypanosome-infected saliva could also be a contributing factor in the decreased platelet aggregation inhibition. Indeed, adenosine deaminases convert adenosine into inosine, a nucleoside that was recently suggested to modulate platelet responses against various agonists including ADP and collagen [36].

The inhibition of the thrombin activity is another key anti-haemostatic activity of normal tsetse saliva. Indeed, a femtomolar affinity thrombin inhibitor (TTI) has been previously characterized in tsetse fly salivary gland extracts and shown to potently inhibit thrombinase activity and thrombin-induced haemostatic reactions [6]. In our study, we demonstrate that the presence of trypanosomes in the salivary glands severely impairs this ability of saliva to inhibit human thrombin and even modifies saliva to enhance the activity of this thrombinase in an *in vitro* pNA-release assay. This observed increase in thrombin activity was not related to the presence of a trypanosome-derived enzyme since SG^+^ saliva by itself did not hydrolyze the chromogenic thrombin substrate (data not shown). Corroborating the observed effects of salivary gland infection on the measured enzymatic activities in the biochemical assays, the anti-coagulant potency of SG^+^ saliva was severely compromised in human plasma coagulation assays using human thrombin as a trigger. Indeed, while all tested SG^-^ saliva dilutions significantly inhibited thrombin-induced coagulation, several SG^+^ saliva dilutions (1/800–1/6400) failed to inhibit this haemostatic reaction and even slightly increased the maximal coagulation response induced by thrombin.

As such, both the biochemical and plasma coagulation assays suggested the presence of a parasite-derived or infection-induced procoagulant factor in the saliva of SG^+^ flies. Known thrombin activity enhancing cofactors include glycoprotein Ibα, fibrin and Na^+^ [37]. Given that experiments were performed under physiological salt conditions (150 mM) with very low saliva concentrations, the influence of Na^+^ ions can be ruled out. Strikingly, tsetse fly transcriptome analyses revealed an abundant representation of a fibrinogen-domain-containing protein family that is enriched in the salivary gland tissue (197 ESTs) as compared to other organs (16 ESTs in midgut, none in the fat body) [38]. Possibly, these or other (tissue or parasite-derived) proteins might modulate thrombin activity through exosite binding and allosteric activation or even contribute as substrate in the coagulation reaction. The possibility that SG^+^ and SG^-^ saliva by itself undergoes coagulation in response to thrombin was excluded experimentally. An experimental approach based on SG^-^/SG^+^ differential salivary proteome analyses and/or affinity purification using thrombin as bait could possibly unveil the identity of this thrombin enhancing factor.

Collectively, we have demonstrated that upon colonization of the tsetse salivary glands with *Trypanosoma brucei*, the protein content and anti-haemostatic activity of the saliva change resulting in an altered insect vector feeding behavior. We assume that the reduced anti-haemostatic activity precludes the SG^+^ tsetse fly from efficiently generating and maintaining a primary blood pool as prerequisite in the feeding process. The observed prolonged probing/feeding time might result in an increased host contact as a result of interrupted feeding and partial blood acquisition and contribute to a higher probability of parasite transmission. To experimentally demonstrate the latter in a natural setting, i.e. to evidence the link between the behavioral modifications of tsetse flies and a more successful parasite transmission, is not obvious. However, field studies have indicated that tsetse flies are highly responsive to host defensive behavior and are prone to interrupted feeding [39]. Given that *T. brucei* salivary gland infected tsetse flies need longer times to feed successfully compared to non-infected ones, this high sensitivity to the host defensive behavior might result in a higher probability of interrupted blood feeding and of alternative host seeking. In other words, it might result in an increased biting rate of the infected tsetse within the available host population. As such, an infected tsetse fly is more likely to probe on multiple hosts during a single feeding cycle. Given that probing alone was proven to be sufficient to infect a mammalian host and that successive probing of the same fly on different hosts results in multiple infections [27], the parasite-induced change in tsetse biting behavior might result in an enhanced trypanosome transmission. Here, it is clear that multiple transmission of the parasite in a single tsetse feeding cycle increases its survival and circulation within the natural mammalian host population. In the case of the human pathogenic *T. brucei* sp., where the numbers of salivary gland infected tsetse flies in the natural population are extremely low [<0.1%, [40]–[42]], the increased biting rate of the infected tsetse could be a major epidemiological factor.

Currently, we do not know the molecular mechanism that underlies the trypanosome-induced modification of saliva composition and biological activities. Possibly, the high density of actively metabolizing parasites causes physiological stress to the salivary gland cells resulting in a suppression of salivary gene transcription and translation. In addition, the significant enhancement of the thrombin activity in the chromogenic thrombinase assay suggests that an activating factor is directly released or induced by the parasites in the saliva.

## Materials and Methods

### Ethics statement

Animal ethics approval for the tsetse fly feeding on live animals and infection with *T.brucei* parasites was obtained from the Animal Ethical Committee of the Institute of Tropical Medicine, Antwerp (Belgium) (Ethical clearance nrs. PAR013-MC-M-Tryp and PAR014-MC-K-Tryp). All tsetse fly infection studies were performed in compliance with the regulations for biosafety and under approval from the Environmental administration of the Flemish government (licencenr. SBB 219.2007/1410).

### Tsetse fly and trypanosome species

Male *Glossina morsitans morsitans* (Westwood) from the colony at the Institute of Tropical Medicine (Antwerp, Belgium) were used in all experiments. This colony originated from pupae collected in Kariba (Zimbabwe) and Handeni (Tanzania) [43]. Flies were fed 4 days per week on rabbits and are maintained at 26°C and 65% relative humidity. Animal ethics approval for the tsetse fly feeding on live animals was obtained from the Animal Ethical Committee of the Institute of Tropical Medicine, Antwerp (Belgium).

The pleiomorphic *Trypanosoma brucei brucei* AnTAR1 strain, derived from the EATRO 1125 stock [44], was used for the infection experiments. This strain was previously demonstrated to develop efficiently in the tsetse fly, resulting in >20% salivary gland infections [45].

### Fly infections

Freshly emerged flies were offered their first blood meal on an anaesthetized mouse showing a pleiomorphic *T. b. brucei* parasitaemia of approximately 10^8^ trypanosomes/ml blood with >80% intermediate/stumpy forms. Only fully engorged flies were further maintained at 26°C and 65% relative humidity and were fed 3 days per week on a naive rabbit. Thirty days after the infective blood meal, individual flies were evaluated for the presence of metacyclic trypanosomes in their salivary glands by salivation on pre-warmed (37°C) glass slides [modification of the method of Burtt *et al*. [46]]. This allowed us to obtain two experimental fly groups of equal age and feeding history but with a different trypanosome infection status in the salivary glands (SG^+^ and SG^-^). These flies were subsequently used for feeding efficiency analysis and for the dissection of salivary glands to assess salivary protein expression and associated biological activities. All tsetse fly infection studies were performed in compliance with the regulations for biosafety and under approval from the Environmental administration of the Flemish government.

### Tsetse fly feeding efficiency

The feeding efficiencies of individualized SG^+^ and SG^-^ flies of the same age and exactly the same feeding history were compared three days after the last blood meal on anaesthetized mice. Feeding efficiencies were monitored by direct observation by two observers (JVDA and GC). Each observer contributed half of the observations for each experimental group, thereby excluding inter-group differences as a result of the different observers. For each fly, the total probing and feeding time was measured with a chronometer (accuracy of 1 sec) by direct observation. In order to determine the blood meal size, individual fly masses were measured to an accuracy of 0.1 mg before and immediately after blood feeding using an analytical balance (Sartorius) as described previously [3].

### Tsetse fly salivary gland and saliva harvest

Three days after the last blood meal and following a 10 minute cold shock at 4°C, salivary glands of SG^+^ and SG^-^ flies were dissected, pooled by 3 pairs in 30 µl sterile physiological H_2_O and incubated on ice for two hours before centrifugation (500 ×*g*, 2 min at 4°C). The supernatants were centrifuged an additional time to obtain saliva devoid of trypanosomes (2500 ×*g*, 2 min at 4°C). Saliva samples were stored at −80°C and only thawed once for analysing protein content and enzymatic activities. SG^-^ and SG^+^ samples were always handled and tested in parallel in all subsequent analyses. Pellets (salivary gland tissue) were further processed to extract RNA for RT-qPCR purposes.

### Quantitative reverse transcription-PCR (RT-qPCR) analysis on a selection of major *G. m. morsitans* saliva genes

The harvested salivary gland tissue was homogenised with a Teflon pestle in 1 ml Tripure reagent (Roche) followed by total RNA extraction according to the manufacturer's protocol. Six-hundred nanogram of each RNA sample was used for primary cDNA synthesis using 100 pmol oligo(dT)_15_ primer (Promega) and 10 units Transcriptor Reverse Transcriptase (Roche). For transcript-analysis, we made a selection of genes based on (i) the available literature data on identified genes that encode soluble saliva proteins, (ii) their relative contribution to tsetse fly proteome in terms of abundance and (iii) their predicted involvement in the blood feeding physiology. According these criteria we selected the identified thrombin inhibitor (TTI), a highly abundant allergen (TAg5), two putative adenosine deaminases (TSGF1&2) that might modulate adenosine-mediated platelet responses, two highly abundant putative endonucleases (Tsal1&2) that might contribute to the blood feeding process by producing a defibrotide-like mixture of DNA haptamers and one predicted and one confirmed apyrase (Sgp3 and 5′Nuc related protein). Relative transcript quantification was performed on an iCycler iQ detection system (Bio-Rad) and using the Bio-Rad software version 3.1. RT-qPCR was performed on triplicate samples in a 25 µl reaction volume, containing 1.5 to 15 ng primary cDNA (depending on the gene), 12.5 µl of iQ SYBR Green Supermix (Bio-Rad) and an optimized primer pair concentration for one of the respective saliva genes: *tti* [500 nM TTI_FW (5′- TTTATCTGATAGTTGCCGCAC -3′) and TTI_REV (5′- AAAGCCTTATGCCAGGAATC -3′)], *tag5* [300 nM TAg5FW (5′-GTGGGTTGTGCCGCTTCTG-3′) and TAg5REV (5′-TTGACCTCGTATTTCTCGTTGG-3′)], *tsal1* [700 nM Tsal1FW (5′-CTGATACCTCGATGATCACTC-3′) and Tsal1REV (5′-AGGCTCTTACATAATCCTTAAC-3′)], *tsal2* [500 nM Tsal2FW (5′-CCAAGAACTGGCTGACCAA-3′) and Tsal2REV (5′-CTGCCAGCAGATTGTGTAAC-3′)], *tsgf1* [300 nM TSGF1_FW (5′-CGGTTGTAAATCCGAATCTGT-3′ and TSGF1_REV (5′-GCGGCTGGCAAATAATGTAGA-3′)], *tsgf2* [500 nM TSGF2_FW (5′-CAAACGCTCCGGTGTTGACGT-3′) and TSGF2_REV (5′-GCGGCTGGCAAATAATGTAGA-3′)], *5*′*nuc* [300 nM 5NucFW (5′-CGGGTAATAAAGTTCTGGTCGTA-3′) and 5NucREV (5′-TTGGCAAGTCCACATTTGTTCTC-3′)] and *sgp3* [500 nM Sgp3_FW (5′- GCTATGGAACCATGGAAGGA -3′) and Sgp3_REV (5′- TTCTGATTCGCCTTCGTCTT -3′)]. For normalization, *G. m. morsitans* actin and tubulin genes were amplified using respectively 700 nM and 300 nM of the following primer pairs: actinFW (5′-CGCTTCTGGTCGTACTACT-3′) and actinREV (5′-CCGGACATCACAATGTTGG-3′), tubulinFW (5′-GATGGTCAAGTGCGATCCT-3′) and tubulinREV (5′-TGAGAACTCGCCTTCTTCC-3′). The PCR conditions comprised an initial 10 min polymerase activation at 95°C followed by 35 cycles, each consisting of a denaturation step at 95°C for 15 s, 60 s annealing at 60°C and 60 s elongation at 72°C. In the data analysis, both actin and tubulin housekeeping genes were included to calculate an integrated normalization factor using the geNorm software v. 3.5.

### Saliva protein analysis

Protein concentrations in the saliva extracts were determined using the BCA protein assay reagent kit (Pierce Biotechnology). Saliva samples of SG^-^ and SG^+^ flies were analyzed by Tricine-SDS-PAGE, using Novex tricine gels 10–20% (1 mm/10 well, Invitrogen) and 100 mM Tris pH 8.3 100 mM Tricine 0.1% SDS as running buffer. Gels were run at 125 V in an XCell Surelock Mini-Cell (Invitrogen). In parallel, the prestained PageRuler protein ladder and Spectra Multicolor Low Range Protein Ladder (Fermentas) were applied to the gels. Gels were either stained with 0.025% Coomassie dye R-250 in 10% acetic acid according to an established protocol [47] or Silverstained using the PageSilver kit (Fermentas) after a 30 minute fixation in 5% glutaraldehyde. The different Coomassie-stained protein profiles were digitalised as 300 dpi greyscale TIFF-files and analysed with the ImageMaster 1D Elite 3.01 programme (Amersham Pharmacia Biotech). In this analysis, the size and intensity of each protein band was quantified by densitometry and expressed as integrated peak density values representing the amount of protein in the respective band.

Different biological activities in tsetse saliva that were previously demonstrated or that could be predicted by EST-database analysis were assayed: thrombin-inhibitory (TTI), apyrase (5′nucleotidase, gmmspg3) and adenosine deaminase activity (TSGF1/2).

### Apyrase activity measurement

Salivary apyrase activity was quantified by assessing the dephosphorylation rate of 20 µM ATP and ADP at 27°C in a 25 mM Tris/HCl pH 7.8 buffer supplemented with 2.5 mM CaCl_2_. ATP/ADP-conversion was monitored after 1 hour by quantifying the release of inorganic phosphate (P*_i_*) using the Malachite green phosphate assay kit (Gentaur) and O.D. measurement (λ = 650 nm) in an Multiskan Ascent microplate reader (ThermoScience). The ATPase and ADPase activities in tsetse saliva samples were expressed as pmole P*_i_* release/min × salivary gland.

### Adenosine-deaminase (ADA) activity measurement

Adenosine deaminase activity in tsetse saliva samples was measured spectrophotometrically by a direct kinetic assay, monitoring the change in O.D. (λ = 265 nm) upon conversion of adenosine into inosine. This ADA activity assay was performed in 10 mM HEPES 150 mM NaCl buffer (pH 7.5) containing 100 µM adenosine and O.D. values were recorded at 15 s interval over a period of 5 min in a microplate reader (ThermoScience). The ADA activity in the saliva samples was expressed as milliUnits ADA/salivary gland, where 1 Unit ADA will deaminate 1 µmole of adenosine to inosine per minute at pH 7.5 (millimolar extinction coefficient of adenosine at 265 nm = 8.1).

### Anti-thrombin activity measurements

The thrombin inhibitory potential of saliva (1/80–1/400 dilution) was quantified in 96-well plates by a kinetic assay at 37°C that monitors the release of p-nitroanilide (pNA) from 750 nM of thrombin chromogenic substrate (β-Ala-Gly-Arg-p-nitroanilide diacetate, Sigma) by the proteolytic activity of human thrombin (Roche, 500 mU/ml) in PBS. pNA-release was measured for at least 1 hour at λ = 405 nm in a microplate reader (ThermoScience). The thrombin inhibitory potential of the saliva samples was expressed relative to the pNA release obtained with thrombin (100% activity).

### Anti-platelet aggregation assay

The platelet aggregation was monitored in a 96-well flat-bottom microplate assay as described elsewhere [48]. Platelet-rich plasma (PRP) was prepared from venous human blood that was anticoagulated in Monovette coagulation tubes (Sarstedt). Aggregation of platelets was induced at 37°C with 10 µM ADP (in 150 mM NaCl) in the presence or absence of serial saliva dilutions (1/100–1/400, in 150 mM NaCl) from SG^+^ and SG^-^ flies. Reduction in optical density (increase in transmission) at 650 nm wavelength was monitored as a measure for platelet aggregation.

### Anti-coagulation assay

Human platelet-poor plasma (PPP), prepared by pelleting the platelets in PRP (see above) at 1500 ×*g* for 15 min, was used for thrombin-induced coagulation assays. Briefly, coagulation was triggered in a total volume of 180 µl by the 1/3 addition of PPP to 10 mM HEPES (pH 7.4) 12.5 mM CaCl_2_ supplemented with thrombin at a 25 mU/ml final concentration in the presence or absence of 1/50–1/6400 dilutions of saliva from SG^+^ and SG^-^ flies. Coagulation was measured as a steep increase in absorbance (λ = 405 nm). The lag phase preceeding coagulation onset was determined as a measure for anti-coagulation activity in the respective saliva samples.

### Genbank accession numbers

*tsal1* (AF259958), *tsal2* (EF409243), *tsgf1* (AF140521), *tsgf2* (AF140522), *5nuc* (AF384674), *sgp3* (EF398273), *tag5* (AF259957), *tti* (AF054616).
